# Impact of Hydroxytyrosol-Rich Extract Supplementation in a High-Fat Diet on Gilthead Sea Bream (*Sparus aurata*) Lipid Metabolism

**DOI:** 10.3390/antiox13040403

**Published:** 2024-03-27

**Authors:** Sara Balbuena-Pecino, Manel Montblanch, Enrique Rosell-Moll, Verónica González-Fernández, Irene García-Meilán, Ramon Fontanillas, Ángeles Gallardo, Joaquim Gutiérrez, Encarnación Capilla, Isabel Navarro

**Affiliations:** 1 Departament de Biologia Cellular, Fisiologia i Immunologia, Facultat de Biologia, Universitat de Barcelona, 08028 Barcelona, Spain; sbalbupe7@alumnes.ub.edu (S.B.-P.); mmontblanch@ub.edu (M.M.); kikerosell@ub.edu (E.R.-M.); vgonzalezf@ub.edu (V.G.-F.); irene.garcia@ub.edu (I.G.-M.); mgallardo@ub.edu (Á.G.); jgutierrez@ub.edu (J.G.); ecapilla@ub.edu (E.C.); 2 Skretting Aquaculture Research Centre, 4016 Stavanger, Norway; ramon.fontanillas@skretting.com

**Keywords:** aquafeeds, olive polyphenols, high-fat diet, lipid metabolism, feed additive, *Sparus aurata*

## Abstract

High-fat diets (HFDs) enhance fish growth by optimizing nutrient utilization (i.e., protein-sparing effect); however, their potential negative effects have also encouraged the search for feed additives. This work has investigated the effects of an extract rich in a polyphenolic antioxidant, hydroxytyrosol (HT), supplemented (0.52 g HT/kg feed) in a HFD (24% lipid) in gilthead sea bream (*Sparus aurata*). Fish received the diet at two ration levels, standard (3% of total fish weight) or restricted (40% reduction) for 8 weeks. Animals fed the supplemented diet at a standard ration had the lowest levels of plasma free fatty acids (4.28 ± 0.23 mg/dL versus 6.42 ± 0.47 in the non-supplemented group) and downregulated hepatic mRNA levels of lipid metabolism markers (*ppara*, *pparb*, *lpl*, *fatp1*, *fabp1*, *acox1*, *lipe* and *lipa*), supporting potential fat-lowering properties of this compound in the liver. Moreover, the same animals showed increased muscle lipid content and peroxidation (1.58- and 1.22-fold, respectively, compared to the fish without HT), suggesting the modulation of body adiposity distribution and an enhanced lipid oxidation rate in that tissue. Our findings emphasize the importance of considering this phytocompound as an optimal additive in HFDs for gilthead sea bream to improve overall fish health and condition.

## 1. Introduction

In comparison with terrestrial animals, the metabolism of aquatic animals has adapted to use lipids, rather than carbohydrates, as the primary source of energy [1]. Thus, fats along with proteins are the two most important constituents in the composition of fish diets. However, protein is a limiting component since it is a costly one [2]. For this reason, a wider use of high-fat diets (HFDs) in numerous farmed fish species has been observed in recent years in order to partially substitute the protein source to economize while, at the same time, trying to fulfill the increasing current demand for fish for human consumption [3]. Indeed, it has been demonstrated that HFDs, with an appropriate composition and level of dietary fat, can enhance growth performance, improving the use of dietary protein (protein-sparing effect), and reproductive traits, throughout a short-term feeding [4]. However, an extra amount of lipids in the diet can also induce adverse effects in fish, leading to compromised mitochondrial and peroxisomal fatty acid β-oxidation efficiency and, consequently, depressed lipid catabolism and increased fat accumulation in the liver, among others [5,6]. Moreover, fat deposition in the liver (or other tissues) can increase lipid peroxidation rate and trigger oxidative stress, inducing tissue damage [7]. Apart from this, it is recognized that the overall lipid content in the diet can also influence feed intake. A reduction in this parameter can be observed in fish fed a HFD, likely due to elevated circulating fatty acid levels and/or larger lipid reserves that may influence hypothalamic anorexigenic centers/neurons [8].

Recently, the protective and ameliorative effects of various dietary feed additives have been investigated as a strategy to mitigate the negative consequences of HFDs in the aquaculture sector (reviewed by [4]). These additives are included in functional diets at very low doses, and most times contribute, due to their origin, to a circular economy, therefore, being affordable strategies from an economic point of view. Among these, 3,4-dihydroxyphenylethanol (i.e., hydroxytyrosol, HT) is a polyphenol that has garnered considerable interest in the past decade for its numerous beneficial bioactive properties in mammals [9]. This natural compound is found in the leaves or fruit of the olive tree (*Olea europaea*), and also in olive oil or juice and their by-products [10]. Mammalian studies have shown that, in addition to its well-known antioxidant capacity, HT plays a protective role against the accumulation of fat [11]. In particular, HT-rich extracts or HT supplementation have been shown to reduce abdominal or hepatic fat deposition, ameliorate morphological alterations of white adipose tissue, prevent inflammation, and decrease the circulating levels of total cholesterol and triglycerides (TG) in HFD-fed rodents [12,13,14,15,16]. In addition, in vitro studies with primary human omental preadipocyte cells and the 3T3-L1 cell line have revealed that HT also inhibits differentiation of preadipocytes, enhances lipolysis, and triggers apoptosis in maturing adipocytes [17,18]. Concerning fish, the anti-obesogenic potential of HT remains largely unexplored. However, in blunt snout bream (*Megalobrama amblycephala*), TG content of primary hepatocytes (treated with oleic acid to induce lipid accumulation) was decreased by HT in a dose-dependent manner (2.5, 5 and 10 µM) [19]. In the same species, but from an in vivo perspective, a HFD (15% lipid) with 100 mg/kg HT supplementation reduced hepatic fat deposition [19], and the same diet but with 200 mg/kg HT reduced reactive oxygen species (ROS) content and mitochondrial dysfunction in the same tissue [20]. Moreover, in a previous study by our group, a decrease in head and viscera adiposity was observed in zebrafish (*Danio rerio*) larvae exposed to 100 µM HT in the water [21]. Within the same study, in primary cultured rainbow trout (*Onchorynchus mykiss*) adipocytes, HT was able to offset the adipogenic effect caused by rosiglitazone, an agonist of the peroxisome proliferator-activated receptor (PPAR) gamma [21]. 

In this framework, the present work aimed to assess the potential anti-obesogenic and lipid metabolism regulatory effects of an HT-rich extract derived from olive juice in juveniles of gilthead sea bream fed a HFD. This study expands on a previously published work [22], wherein growth performance in the same experimental animals was evaluated by means of plasma and gene expression levels of related molecules (from the growth hormone/insulin-like growth factor (IGF) axis, for instance). In that study, the use of HT as a dietary additive seemed to induce an improved growth potential and an anabolic muscle condition in this fish species.

## 2. Materials and Methods

### 2.1. Experimental Trial

Juveniles of gilthead sea bream (*Sparus aurata*) were purchased from a Mediterranean hatchery (Piscimar, Burriana, Spain). In the fish facilities of the Faculty of Biology at the University of Barcelona (UB, Barcelona, Spain), fish were kept in a semi-closed seawater recirculation system at optimal conditions for this species (for details see [22]). During the one-month acclimation period, the animals were fed *ad libitum* at 9 a.m. and 4 p.m. with a commercial diet (18% lipids, 48.5% protein and 18.5 MJ/kg digestible energy) (OptiBream AE Skretting, Burgos, Spain). After this period, the fish were weighed and randomly distributed into eight 200 L (n = 15 fish/tank) and four 400 L (n = 30 fish/tank) tanks at the same biomass density (4 kg·m^−3^). Next, fish were provided *ad libitum* access to the experimental HFD without the HT-rich extract for 1 week. This served the dual purpose of habituating the fish and visually identifying the ration corresponding to satiation, which was then set as the standard during the trial. 

The 8-week in vivo experiment was run from the end of August until the end of October and performed as previously explained [22]. The initial body weight and stock density of the fish were 80.81 ± 1.43 g and 6 kg·m^−3^. The experimental HFD, with a lipid content of 24% of the dry matter, 46.8% protein and 23 MJ/kg of digestible energy, was developed and manufactured by Skretting Aquaculture Research Center (Stavanger, Norway). This diet included 50% fish oil and 50% rapeseed oil (refer to Table 1 for details). Additionally, the diet was formulated without (HF) or with an HT-rich extract (HF+HT), at a concentration of 0.52 g of HT per kg of feed, and was administered daily at two different ration levels: standard (3% of the total fish weight in the tank) (ST) or restricted (40% reduction) (RE). Each experimental group included sets of three replicate tanks (one of 400 L and two of 200 L, due to the particular tank distribution at the UB fish facility). The olive juice extract rich in HT incorporated into the formulation, named HIDROX^®^ and supplied by Oliphenol LLC. (Hayward, CA, USA), contained over 12% of both simple and total polyphenols, specifically 3.136% HT, 0.216% oleuropein, and 0.408% tyrosol (certificated analysis number 12-190403-000). The selection of the dose of HT was already explained in Balbuena-Pecino et al. [22]. All groups of fish were given 60% of their ration in the morning, and those receiving the standard one were fed the remaining 40% in the afternoon. The daily ration was tuned up according to body weight every 2 weeks. The main trial spanned 8 weeks, with certain fish kept for an extra week to perform the histological analysis and in vitro experiments at week 9.

Before sampling, the fish underwent a 24-h fasting period to minimize the risk of sample contamination from the digestive tract contents. After 8 weeks, all animals were anesthetized with MS-222 (E10521, Sigma-Aldrich, Tres Cantos, Spain) and weighed. This comprised 41 fish from HF_ST, 48 from HF_RE, 38 from HF+HT_ST, and 48 from HF+HT_RE. Then, 10 random animals per group were sampled. Blood was drawn from the caudal vessels using EDTA-Li, and the plasma obtained after centrifugation was stored at −20 °C until further analysis. Following sacrifice through spinal cord sectioning, the liver and visceral adipose tissue were dissected and weighed to calculate hepatosomatic and mesenteric fat (MFI) indices, respectively. Small pieces of liver, adipose tissue, and white muscle were then rapidly frozen in liquid nitrogen and preserved at −80 °C until use. Furthermore, at week 9, samples of liver and visceral adipose tissue from six additional individuals per group were taken for histological analysis. All procedures related to the handling of animals adhered to the guidelines outlined in the European Union Council directive (EU 2010/63) and were approved by the Ethics and Animal Care Committee of UB with reference numbers CEEA 34/20 and DAAM 11251.

### 2.2. Somatic Parameters and Lipid Composition

Somatic parameters were determined at week 8. Most of them were presented in [22], except for the MFI index, which was calculated per fish [(mesenteric fat weight/body weight) × 100] and then averaged per each tank (n = 3).

To assess the lipid content, 300 mg of liver and 700 mg of white muscle were pulverized with liquid nitrogen in a mortar, and then extracted with a 2:1 chloroform:methanol solution following the method of Bligh and Dyer [23]. Next, the lipid extracts were allowed to dry, and the total lipid was determined gravimetrically and expressed as a percentage (n = 10).

### 2.3. Plasma Metabolites

The circulating plasma levels of glucose (41011, Spinreact, Sant Esteve d’en Bas, Spain), free fatty acids (FFA) (MAK044, Sigma-Aldrich, Tres Cantos, Spain), high-density lipoproteins (HDL) and low-/very-low density lipoproteins (LDL/VLDL) (MAK045, Sigma-Aldrich, Tres Cantos, Spain), as well as TG (41030, Spinreact, Sant Esteve d’en Bas, Spain), were measured at week 8. The metabolites were determined by enzymatic colorimetric methods following the manufacturers’ instructions (n = 10).

### 2.4. Histology

After 9 weeks of experimental trial, liver and visceral adipose tissue samples were fixed with 10% *v*/*v* formalin for 1 week, dehydrated through a graded series of ethanol (70%, 80%, 90%, and absolute), and paraffin-embedded. Paraffin sections of 7 μm thickness were acquired using a rotary microtome (Rotary 3003 PFM microtome, Köln, Germany), stained with hematoxylin and eosin (H&E), and then cover-slipped with DPX (Sigma-Aldrich, Tres Cantos, Spain). Next, preparations were examined under a light microscope at 10× magnification, and images were captured with the Olympus PM10SP Automatic Photomicrography System. The percentage area occupied by lipid droplets within the hepatocytes in liver sections, as well as the area and number of adipocytes in adipose tissue preparations, were determined by analyzing three images captured from various tissue sections of each fish (n = 6) using ImageJ software v. 1.52n (National Institutes of Health, Bethesda, MD, USA).

### 2.5. Gene Expression

#### 2.5.1. RNA Extraction and cDNA Synthesis

Tissue homogenates were obtained from 30 mg of liver and 100 mg of adipose tissue or white muscle with 1 mL of TRI Reagent^®^ using the Precellys^®^ Evolution homogenizer, cooled with Cryolys^®^ (Bertin Technologies, Montigny-le-Bretonneux, France). Subsequently, RNA was extracted following the protocol of the TRI Reagent^®^ manufacturer, and resuspended in DEPC water. RNA concentration and purity were assessed using a NanoDrop2000 spectrophotometer (Thermo Scientific, Alcobendas, Spain), and integrity was confirmed through electrophoresis on a 1% (*w*/*v*) agarose gel stained with 3% SYBR Safe DNA gel stain (Bio-Rad, El Prat de Llobregat, Spain). Then, 2000 ng of RNA underwent DNase I treatment (Life Technologies, Alcobendas, Spain), and was finally retro-transcribed with the Transcriptor First Strand cDNA Synthesis Kit (Roche, Sant Cugat del Vallès, Spain).

#### 2.5.2. Real-Time Quantitative PCR (qPCR)

Quantitative gene expression data were obtained adhering to the MIQE guidelines [24] with a CFX384^TM^ Real-Time System (Bio-Rad, El Prat de Llobregat, Spain). cDNA samples were evaluated in triplicate using iTaq Universal SYBR^®^ Green Supermix (Bio-Rad, El Prat de Llobregat, Spain), as previously detailed by Balbuena-Pecino et al. [25]. The assessed genes included the following: the transcription factors ppar alpha (*ppara*), beta (*pparb*) and gamma (*pparg*); the fatty acid uptake and transport markers lipoprotein lipase (*lpl*), cluster of differentiation 36 (*cd36*), long-chain fatty acid transport protein 1 (*fatp1*), and fatty acid binding protein 1 (*fabp1*); the peroxisomal and mitochondrial β-oxidation markers acyl-CoA oxidase 1 (*acox1*), carnitine palmitoyltransferase 1a (*cpt1a*), hydroxyacil-CoA dehydrogenase (*hadh*); and the lipolytic and lipogenic enzymes fatty acid synthase (*fasn*), adipose triglyceride lipase (*atgl*), hormone-sensitive lipase or lipase E (*lipe*) and lipase A (*lipa*). The expression level of the genes of interest was determined in relation to the geometric mean of the two reference genes with the highest stability (ribosomal protein s18 (*rps18*) and elongation factor 1 alpha (*ef1a*)), following [26]. Reference gene stability and the relative expression of target genes were assessed with CFX Manager 3.1 software. Sequences, annealing temperatures, and accession numbers of the primers used can be found in Supplementary Table S1.

### 2.6. Protein Homogenates and Western Blotting

Proteins were collected from 30 mg of liver or 100 mg of white muscle in 1 mL of RIPA buffer, with protease (#05892970001, Roche, Sant Cugat del Vallès, Spain) and phosphatase (#04906837001, Roche, Sant Cugat del Vallès, Spain) inhibitor cocktail tablets. Homogenization was performed with the Precellys^®^ Evolution Super Homogenizer coupled to Cryolys^®^ at 4 °C (Bertin Technologies, Montigny-le-Bretonneux, France). After 30 min at 4 °C in a rotating shaker and a centrifuge at 16,000× *g* for 15 min, supernatants were collected, and the amount of protein was measured by the Bradford method [27]. Equal amounts of protein from each sample were mixed with Laemmli buffer (containing β-mercaptoethanol) and heated at 95 °C for 5 min. Twenty or 40 µg of sample from liver or white muscle, respectively, were subjected to 12% SDS-PAGE at 100 V for ~2 h and transferred to Immobilon^®^-FL polyvinylidene fluoride membranes (IPFL00010, Merck Millipore, Cork, Ireland) at 100 mA for another ~2 h. At this point, the transferred protein was quantified by a 5-minute incubation with Revert^TM^ 700 Total Protein Stain solution (#926-11015, LI-COR Inc., Servicios Hospitalarios, Barcelona, Spain), and the signal was detected in the 700 nm channel of the Odyssey^®^ Fc Imaging System (LI-COR Inc., Lincoln, NE, USA). Following a wash, membranes were blocked with Intercept^®^ Blocking buffer (TBS) (#927-50000, LI-COR Inc., Servicios Hospitalarios, Barcelona, Spain) at room temperature for 1 h and incubated overnight at 4 °C with rabbit monoclonal anti-CD36 (D8L9T) primary antibody (1:500, #14347, Cell Signaling Technology, Beverly, MA, USA) diluted in the same blocking buffer-TWEEN^®^ 20 0.1%. After washing several times with TBS-TWEEN^®^ 20 0.1% and TBS, membranes were incubated with an IRDye^®^ 800CW goat anti-rabbit IgG secondary antibody (1:5000, #925-32211, LI-COR Inc., Servicios Hospitalarios, Barcelona, Spain) diluted in the same blocking buffer-TWEEN^®^ 20 0.1% at room temperature for 1 h. Next, membranes were rewashed again, and the signal was detected in the 800 nm channel. Finally, the intensity of bands was quantified using Image Studio^TM^ v. 5.2 software, and the target protein levels of each sample were normalized to their corresponding total protein amount (n = 4 for liver and n = 5 for white muscle).

### 2.7. Lipid Peroxidation

Malondialdehyde (MDA) and 4-hydroxynonenal (4-HNE) levels were measured in liver and white muscle samples as oxidative stress biomarkers for lipid peroxidation. The concentration of these biomarkers was determined using a commercial kit (KB030002, Bioquochem, Gijón, Spain) following the manufacturer’s instructions (n = 10). The level of lipid peroxidation is expressed as the amount of MDA + 4-HNE (nmol) per mg of protein, with the soluble protein concentrations used for the normalization determined using the Bradford method [27].

### 2.8. Preadipocyte Primary Culture and Treatments

Primary cultures of preadipocytes were performed at week 9 following the procedure previously established by Salmerón et al. [28] using visceral adipose tissue from a single fish. Four independent culture replicates were performed per dietary group (HF and HF+HT at standard ration). Cells were plated at a final density of 4.3 × 10^4^ cells/cm^2^ in 1% gelatin pre-treated 12-well plates in growth medium (GM) composed of Dulbecco’s Modified Eagle’s Medium (DMEM)-high glucose (D7777, Sigma-Aldrich, Tres Cantos, Spain), supplemented with 10% fetal bovine serum, 1% antibiotic-antimycotic solution (A5955, Sigma-Aldrich, Tres Cantos, Spain), and 60 mM NaCl, at 23 °C and 2.5% CO_2_. Medium was changed at 2-day intervals. Upon reaching confluence (day 8), cells coming from either HF or HF+HT groups were induced to differentiate by adding 5 µL/mL of lipid mixture (L5146, Sigma-Aldrich, Tres Cantos, Spain) to the GM, in the absence or presence of pure HT (70604, CAS No. 10597-60-1, Cayman chemicals, Ann Arbor, MI, USA) at concentrations of 10 or 100 µM for 72 h. HT was dissolved in ethanol, and cells without HT were treated with the same amount of vehicle (0.1% ethanol). Additionally, cells were incubated without the lipid mixture and used as a negative control of differentiation.

#### 2.8.1. Cell Viability

To evaluate cell viability, the methylthiazolyldiphenyl-tetrazolium bromide (MTT) method was used, as previously reported elsewhere [29], in adipocyte samples from duplicate wells of 12-well plates. During the last 18 h of the total 72 h treatment, cells were incubated with a final concentration of 0.5 mg/mL of MTT (M5655, Sigma-Aldrich, Tres Cantos, Spain), washed with PBS, and resuspended in dimethyl sulfoxide for 3 h. Viability values were calculated from the absorbance readings at 570 nm, corrected at 650 nm, using a microplate reader (Tecan Infinite M200, Männedorf, Switzerland) (n = 4 independent cultures).

#### 2.8.2. Lipid Accumulation

Intracellular neutral lipid content was determined by means of Oil Red O (ORO) staining, as performed in previous studies with the same cell type and species [30]. In brief, after 72 h treatment, adipocytes from duplicate wells in 12-well plates were fixed with 10% formalin and stained with 0.3% ORO (O0625, Sigma-Aldrich, Tres Cantos, Spain) for 2 h. The dye was eluted in 2-propanol, and absorbance was measured at 490 nm with a microplate reader (Tecan Infinite M200, Männedorf, Switzerland). Then, total protein content was determined by staining the cells with Coomassie brilliant blue G-250 for 1 h, extracting the dye using 85% propylene glycol during 1 h at 60 °C, and measuring absorbance at 630 nm. Lipid accumulation values were calculated as the ratio of lipid absorbance readings relative to the protein absorbance ones (n = 4 independent cultures).

### 2.9. Statistical Analysis

The data were analyzed with IBM SPSS Statistics v. 27 (IBM Corp., Armonk, NY, USA) and presented as mean + standard error of the mean (SEM) using GraphPad Prism v. 7 (GraphPad Software, La Jolla, CA, USA). Tanks were considered biological replicates for somatic parameters, while individual fish served as replicates for all other results. Normality of the data was checked using the Shapiro–Wilk test, and homoscedasticity using Levene’s test. Statistical differences were evaluated by a two-way analysis of variance (two-way ANOVA), with diet (HF or HF+HT) and ration (ST or RE) set as independent factors. For in vitro assays, statistical differences were also assessed by a two-way ANOVA, with diet and treatment set as independent factors. In any case, when a significant interaction between factors was observed, group comparisons were examined using the Tukey’s post hoc test. The *p*-value for statistical significance was set at 0.05 for all analyses.

## 3. Results

### 3.1. Somatic Parameters, Lipid Composition and Plasma Metabolites

After 8 weeks of the feeding trial, the MFI and the lipid content of the liver remained unchanged in response to any factor (Table 2). On the other hand, an interaction between both variables was found in the lipid content of white muscle, which was significantly higher in the animals that received the diet with the HT-rich extract at a standard ration (HF+HT_ST) compared to the ones that received the HF diet at the same ration (Table 2). Regarding plasma metabolites, diet composition and feeding regime, as well as their interaction, had significant effects on FFA levels, which were higher in the HF_ST group in comparison with the other three groups (Table 3). The concentrations of HDL, LDL/VLDL and TG were affected only by feeding regime and were significantly lower under the restricted one, regardless of the diet. Finally, glucose levels remained unaltered (Table 3).

### 3.2. Histological Changes in Liver and Adipose Tissue

**Figure 1:**
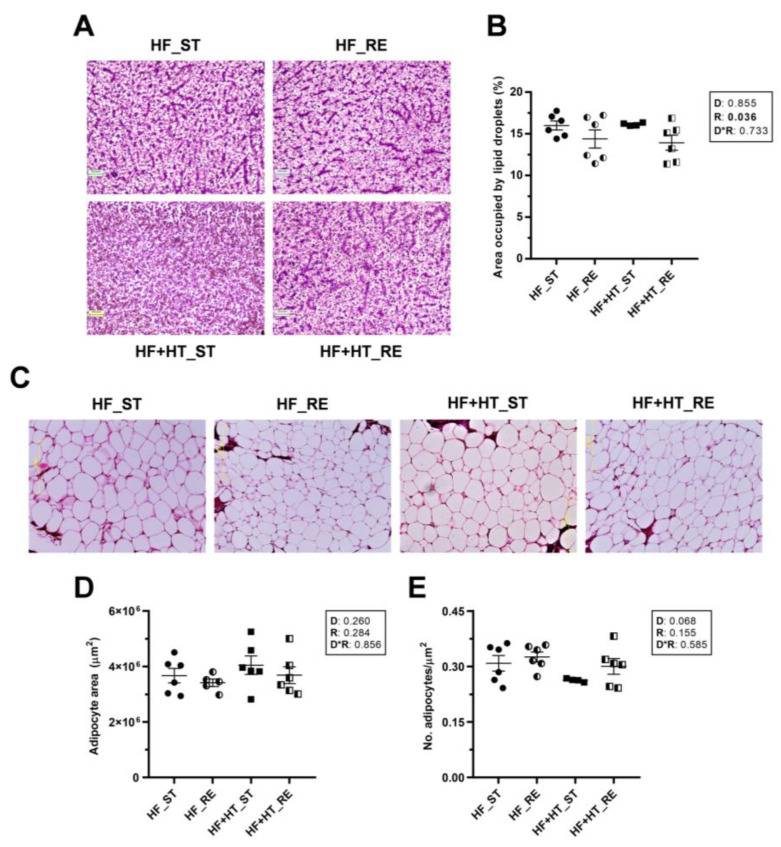
(**A**) Representative images of liver sections stained with hematoxylin and eosin (H&E) and (**B**) percentage area occupied by lipid droplets within hepatocytes. (**C**) Representative images of H&E stained adipose tissue sections, (**D**) area and (**E**) number of adipocytes/mm^2^. Tissue samples are from gilthead sea bream juveniles fed with the experimental diets. A high-fat diet alone (HF) or supplemented with (HF+HT) hydroxytyrosol (0.52 g HT per kg feed) at standard (ST) (3% total fish weight/tank) or restricted ration (RE) (40% reduction) for 9 weeks. Magnification 10×. Data are shown as mean + SEM (n = 6 fish). Statistical differences are indicated in three components: diet (D), ration (R) and interaction (D*R), using two-way ANOVA (*p* < 0.05, shown in bold).

In liver sections, the resulting images and subsequent quantification showed a significant reduction in the percentage area occupied by lipid droplets within the hepatocytes of fish under restricted ration, regardless of the diet (Figure 1A,B). In contrast, adipose tissue slices did not reveal differences in either the area or the number of adipocytes among groups in response to any factor. However, the number of adipocytes in the HF+HT-fed fish at a standard ration was slightly lower than in the other three groups (*p*-value of diet factor = 0.068) (Figure 1C–E).

### 3.3. Lipid Metabolism in the Liver

**Figure 2:**
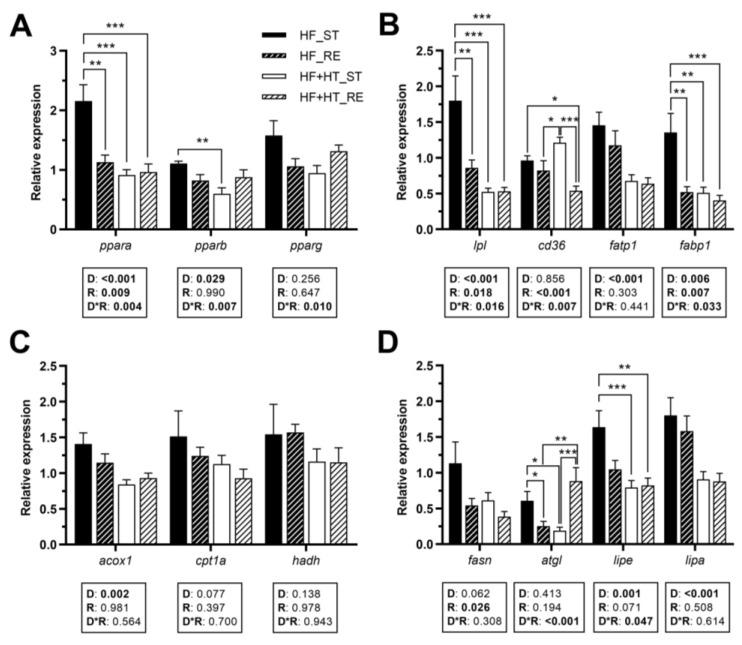
Relative gene expression of (**A**) transcription factors, (**B**) fatty acid uptake and transport markers, (**C**) peroxisomal and mitochondrial β-oxidation markers, and (**D**) key lipolytic and lipogenic enzymes in liver of gilthead sea bream juveniles fed with the experimental diets. A high-fat diet alone (HF) or supplemented with hydroxytyrosol (HF+HT) (0.52 g HT per kg feed) at standard (ST) (3% total fish weight/tank) or restricted ration (RE) (40% reduction) for 8 weeks. Data are shown as mean + SEM (n = 10 fish). Statistical differences are indicated in three components: diet (D), ration (R) and interaction (D*R), using two-way ANOVA (*p* < 0.05, shown in bold). Comparisons among groups were analyzed by a Tukey’s post hoc test when the interaction between the two factors was significant, and significant differences are indicated by asterisks (*p* < 0.05 shown as *; *p* < 0.01 **; *p* < 0.001 ***).

Hepatic gene expression of the transcription factor *ppara* was modulated by diet, feeding regime, and by the interaction of the two factors. *ppara* mRNA levels were higher in the fish of the HF_ST group compared to the other three groups. Similarly, a diet effect, but also an interaction between diet and feeding regime, was found for *pparb*, presenting higher transcript levels in animals fed with HF diet at a standard ration than those fed with the diet containing the HT-rich extract (HF+HT) at the same ration. Furthermore, a significant interaction between the two factors was observed in the case of *pparg* (Figure 2A). 

With regard to fatty acid transporters, diet composition and feeding regime, as well as their interaction, had significant effects on *lpl* and *fabp1* gene expression (Figure 2B). The mRNA levels of *lpl* and *fabp1* were increased in the HF_ST group in comparison with the other groups. Furthermore, gene expression of *cd36* was affected by the ration, and by the interaction of the two factors. Results showed a significant increase in the transcript levels of this gene in the animals of the HF+HT_ST group compared to both groups of fish fed under the restricted regime. Additionally, the mRNA level of *cd36* in the HF_ST group was higher compared to that in the HF+HT_RE group (Figure 2B). The diet effect was found for *fatp1* and the peroxisomal β-oxidation marker *acox1*, presenting downregulated expression in the fish that received the HF+HT diet compared to those that received the HF diet, irrespective of the feeding regime. On the other hand, the mitochondrial β-oxidation markers (*cpt1a* and *hadh*) were not altered in response to any variable (Figure 2C).

The restricted regime, compared to the standard regime, significantly decreased the mRNA levels of *fasn*. An interaction between the two variables was found for the lipase *atgl*, which exhibited the highest mRNA levels in the HF_ST and HF+HT_RE groups compared to the other two. On the other hand, compared to the fish fed with the HF+HT diet, the HF-fed group upregulated the mRNA levels of *lipe* and *lipa*. An interaction between the effects of diet composition and feeding regime was also found for *lipe*, and, as a result, only the standard ration of HF fish caused significant differences compared to both groups of fish fed with the HF+HT diet (Figure 2D).

### 3.4. Lipid Metabolism in Adipose Tissue

**Figure 3:**
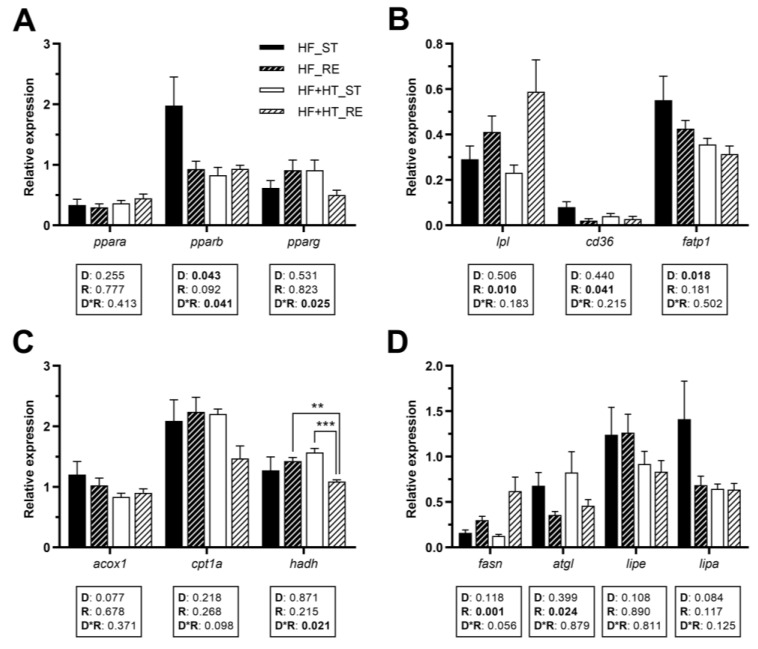
Relative gene expression of (**A**) transcription factors, (**B**) fatty acid uptake and transport markers, (**C**) peroxisomal and mitochondrial β-oxidation markers, and (**D**) key lipolytic and lipogenic enzymes in adipose tissue of gilthead sea bream juveniles fed with the experimental diets. A high-fat diet alone (HF) or supplemented with hydroxytyrosol (HF+HT) (0.52 g HT per kg feed) at standard (ST) (3% total fish weight/tank) or restricted ration (RE) (40% reduction) for 8 weeks. Data are shown as mean + SEM (n = 10 fish). Statistical differences are indicated in three components: diet (D), ration (R) and interaction (D*R), using two-way ANOVA (*p* < 0.05, shown in bold). Comparisons among groups were analyzed by a Tukey’s post hoc test when the interaction between the two factors was significant, and significant differences are indicated by asterisks (*p* < 0.01 shown as **; *p* < 0.001 ***).

In visceral adipose tissue, the gene expression of *pparb* was influenced by both diet composition and its interaction with the feeding regime, while *pparg* was only modulated by the interaction of the two factors. However, none of these factors affected *ppara* gene expression (Figure 3A). Under restricted feeding conditions, *lpl* mRNA levels were significantly higher than in the standard-fed groups. Conversely, the opposite trend was observed for *cd36*, as its mRNA levels were lower under restricted feeding conditions compared to the standard-fed groups. The two groups receiving the HF diet, as opposed to those supplemented with HT, significantly upregulated *fatp1* mRNA levels (Figure 3B). Regarding mitochondrial β-oxidation markers, the gene expression of *hadh* was modulated by the interplay between diet and feeding regime, and its mRNA values were lower in the HF+HT-fed fish at restricted regime in comparison with HF_RE and HF+HT_ST groups. Other than that, no changes were observed in the gene expression of *acox1* and *cpt1a* (Figure 3C). 

The transcript levels of *fasn* and the lipase *atgl* responded to ration. Specifically, the mRNA values of *fasn* were significantly higher in fish subjected to the restricted ration compared to those on the standard one. Conversely, in the case of *atgl*, fish fed the standard ration presented higher mRNA levels than those fed with the restricted ration. On the other hand, there were no statistical differences in *lipe* and *lipa* genes among the different groups of fish (Figure 3D).

### 3.5. Lipid Metabolism in White Muscle

**Figure 4:**
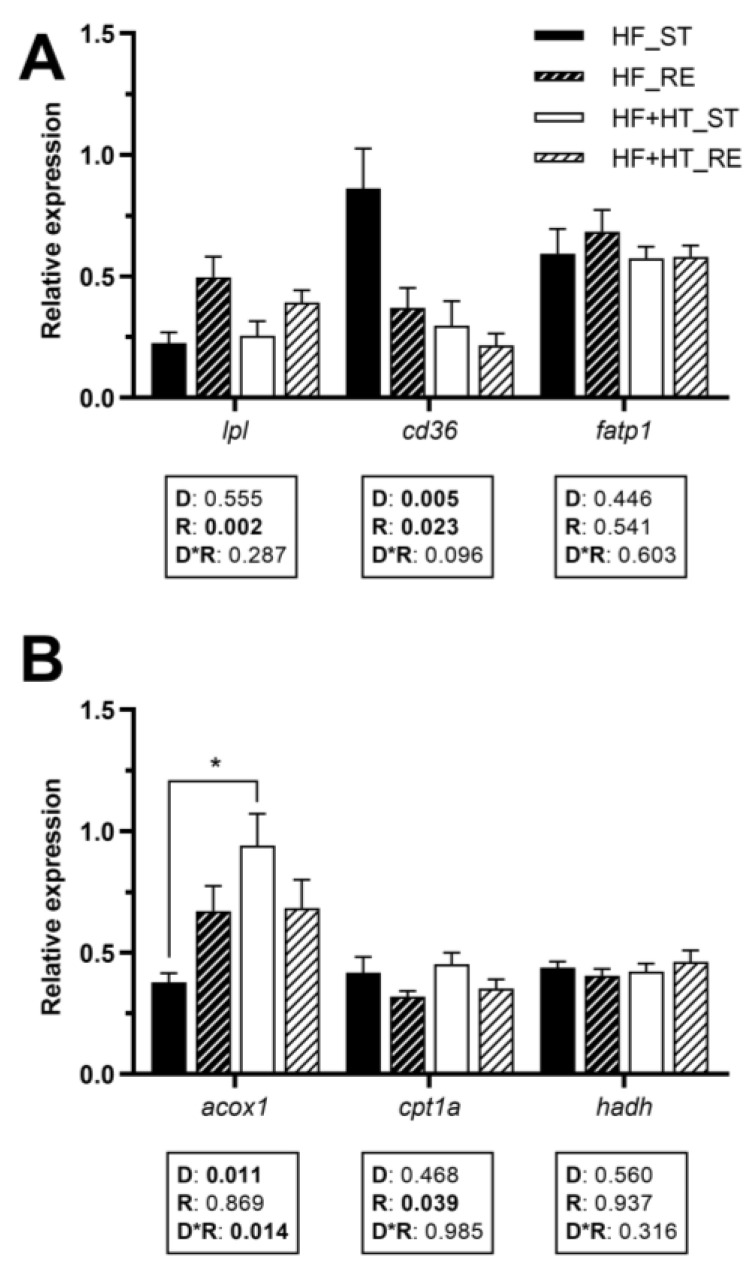
Relative gene expression of (**A**) fatty acid uptake and transport markers and (**B**) peroxisomal and mitochondrial β-oxidation markers in white muscle of gilthead sea bream juveniles fed with the experimental diets. A high-fat diet alone (HF) or supplemented with hydroxytyrosol (HF+HT) (0.52 g HT per kg feed) at standard (ST) (3% total fish weight/tank) or restricted ration (RE) (40% reduction) for 8 weeks. Data are shown as mean + SEM (n = 10 fish). Statistical differences are indicated in three components: diet (D), ration (R) and interaction (D*R), using two-way ANOVA (*p* < 0.05, shown in bold). Comparisons among groups were analyzed by a Tukey’s post hoc test when the interaction between the two factors was significant, and significant differences are indicated by asterisks (*p* < 0.05 shown as *).

The gene expression of *lpl* in white muscle was influenced by the feeding regime, exhibiting upregulation under restricted feeding conditions. A noticeable reduction in *cd36* mRNA levels was detected in fish fed the HF+HT diet compared to those on the HF diet. Additionally, feeding regime also affected this gene, decreasing its gene expression when fish were subjected to the restricted ration. The transcript levels of *fatp1* remained unchanged in response to any factor (Figure 4A). 

The mRNA levels of *acox1* were influenced by the diet composition and its interaction with the feeding regime, and were significantly higher in fish fed the HF+HT diet at the standard ration compared to those fed the HF diet under the same feeding conditions. Regarding mitochondrial β-oxidation markers, the mRNA levels of *cpt1a* were decreased under restricted feeding conditions, regardless of the diet, whereas *hadh* was not affected by any of the factors (Figure 4B).

### 3.6. CD36 Protein Expression and Lipid Peroxidation in Liver and White Muscle

**Figure 5:**
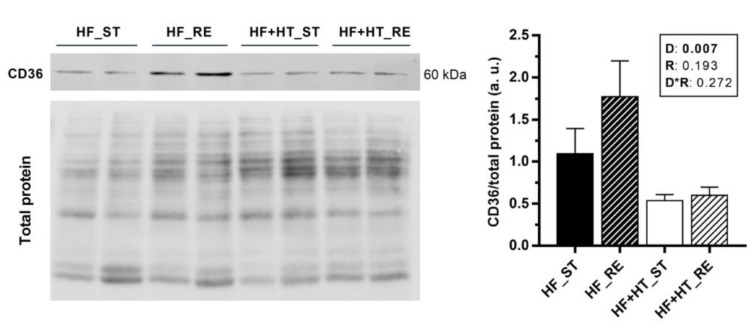
Representative Western blots and quantification of CD36 protein levels normalized to total protein (Revert^TM^) in liver samples of gilthead sea bream juveniles fed with the experimental diets. A high-fat diet alone (HF) or supplemented with hydroxytyrosol (HF+HT) (0.52 g HT per kg feed) at standard (ST) (3% total fish weight/tank) or restricted ration (RE) (40% reduction) for 8 weeks. Data are shown as mean + SEM (n = 4 fish). Statistical differences are indicated in three components: diet (D), ration (R) and interaction (D*R), using two-way ANOVA (*p* < 0.05, shown in bold).

Protein expression of CD36 was higher in the liver of fish fed with the HF diet compared to the ones fed with the HF+HT diet, independently of the ration (Figure 5). In contrast, expression of CD36 could not be detected in white muscle with the antibody and conditions used in the present study.

Diet composition led to increased MDA and 4-HNE levels in the white muscle of fish fed with the HF+HT diet compared to the HF-fed ones, while no differences were observed in the liver (Table 4).

### 3.7. Viability and Lipid Accumulation in Primary Cultured Preadipocytes

**Figure 6:**
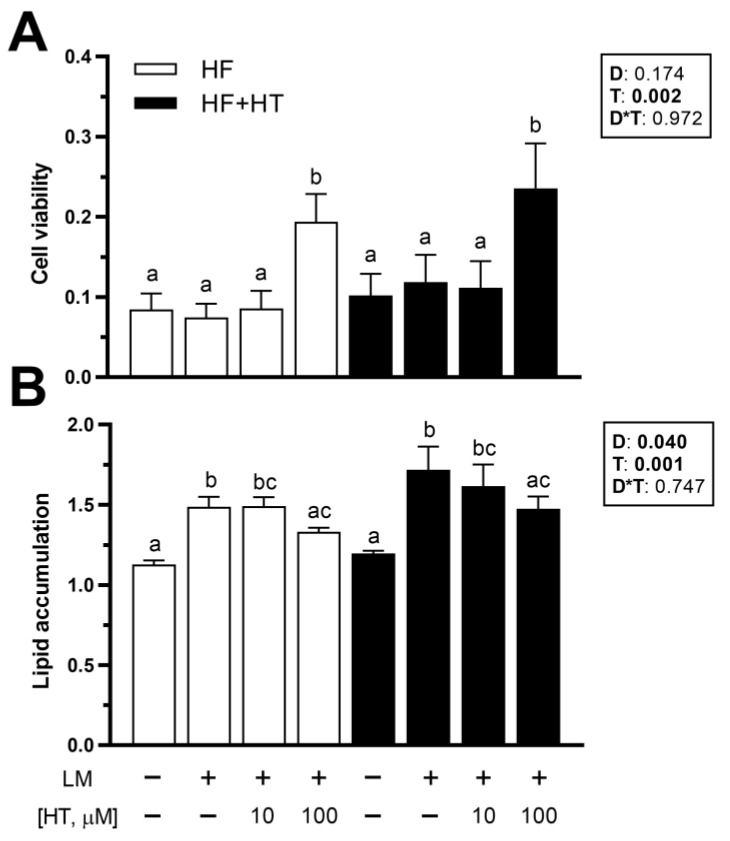
(**A**) Viability and (**B**) quantification of lipid content by Oil Red O staining in adipocytes incubated at day 8 of culture for 72 h with growth media (GM) (0.1% ethanol), GM 5 µL/mL of lipid mixture (LM), or hydroxytyrosol (HT) at different concentrations (10 and 100 µM) + 5 µL/mL of LM. Cells were extracted from gilthead sea bream juveniles fed with the experimental diets. A high-fat diet alone (HF) or supplemented with (HF+HT) HT (0.52 g HT per kg feed), at a standard ration (3% total fish weight/tank) for 9 weeks. Data are shown as mean + SEM (n = 4 independent cultures). Statistical differences are indicated in three components: diet (D), treatment (T) and interaction (D*T), using two-way ANOVA (*p* < 0.05, shown in bold). Comparisons among groups were analyzed by a Tukey’s post hoc test when treatment factor was significant, and significant differences are indicated by letters.

Concerning the in vitro data, cell viability was not affected by the diet factor. After 72 h of exposure, HT 100 µM treatment increased adipocyte viability, whether the cells came from animals fed the HF or HF+HT diets, compared to the other three experimental treatments (Figure 6A). Lipid accumulation was affected by diet composition, as higher values were found in the adipocytes derived from fish that were fed the HF+HT diet. Moreover, this parameter was also modified in response to the different treatments, since the exposure of adipocytes to lipid mixture alone (positive control) and HT at 10 µM concentration + lipid mixture significantly elevated the intracellular lipid content in the cells compared to the control group (GM) in the absence of any treatment. On the other hand, the addition of HT at 100 µM was able to significantly reduce the lipid accumulation induced by the lipid mixture, since the levels of ORO were lower in those cells compared with the GM + lipid mixture treated cells (Figure 6B).

## 4. Discussion

The use of HFDs is currently a prominent trend in aquaculture due to their protein-sparing and growth-enhancing effects [31]. However, an excess intake of dietary lipids can induce unfavorable effects, leading to metabolic alterations such as impaired fatty acid β-oxidation or increased fat accumulation, among others [5,6,7,32]. In this study, the aim was to evaluate the potential of an HT-rich extract derived from olive juice in mitigating the undesirable and adverse effects associated with long-term HFD feeding, specifically focusing on lipid metabolism (uptake, mobilization and oxidation), in gilthead sea bream, an important fish species in Mediterranean aquaculture.

HFDs are known to increase overall adiposity in fish; nonetheless, fat deposition is not evenly distributed across all regions of the body, as it depends on the major site of lipid storage in each species and on the life cycle status of the animal [33]. The perivisceral and subcutaneous adipose tissue depots, together with skeletal muscle and liver, have been identified as the preferential sites for lipid storage in teleost fish [34,35]. Dietary supplementation with key bioactive phytocompounds may play an indirect protective function against the adverse effects of HFDs by lowering or redistributing lipid content among tissues or fat depots. Indeed, previous studies in rodents have demonstrated the potential of HT supplementation in mitigating hepatic fat deposition in animals fed a HFD [12,14,36]. More recently, this effect has also been observed in fish, specifically in blunt snout bream [19] and spotted sea bass (*Lateolabrax maculatus*) [37]. In gilthead sea bream, under the current experimental conditions, the addition of HT to the HFD did not affect the HSI [22], nor did it result in a significant reduction in liver fat content, as indicated by both proximate composition and histological analysis. However, it is worth noting that the HF+HT_ST group exhibited the lowest percentage of hepatic lipid content, suggesting a slight lipid-reducing potential of HT when comparing with the HF_ST group (1.26-fold), in agreement with the lipid-lowering effect previously described in fish liver. Histologically, these changes were not detected, as most of the hepatocytes in all groups were enlarged, with their nuclei displaced towards the periphery, and contained numerous vacuoles in the cytoplasm, showing an intermediate level of fat accumulation according to the scoring system proposed by Ruiz et al. [38] for this same species. Nonetheless, from a quantitative standpoint, the groups of fish fed with a restricted ration showed a significantly lower percentage of hepatic parenchyma occupied by lipid droplets compared to the corresponding standard-fed groups.

In terms of proximate analysis, different from what occurred in the liver, the interaction between diet and feeding regime produced significant effects on lipid deposition in the muscle. Under standard feeding conditions, the muscle lipid content was significantly increased in fish that received the diet incorporating the HT-rich extract compared to those fed without the extract (1.58-fold). These findings are in agreement with a previous work, by Yilmaz et al. [39], that reported increased lipid levels in the muscle of African catfish (*Clarias gariepinus*) with increasing dietary olive pomace oil (9% and 3% concentration), while total lipids in the liver were also diminished. Thus, gilthead sea bream shows a response similar to that of the African catfish concerning the effects of HT dietary supplementation in muscle lipid content, while in the studies in blunt snout bream [19] and spotted sea bass [37], muscle lipid content was not considered. Altogether, these results support the beneficial ability of dietary HT to protect against hepatic fat accumulation but, at the same time, indicate that the use of HT as a feed additive might increase muscle lipid content, which can influence sensory properties, flesh quality, and the overall nutritional value of the fish [1]. Nevertheless, although changes in muscle composition may have marketing consequences, small alterations or increases in the percentage of lipid content are more noticeable in fish species that do not store fat in their muscles, such as turbot (*Psetta maxima*) [40].

Regarding lipid accumulation in distinct adipose tissue depots and the potential anti-obesogenic effect of HT, this polyphenol significantly decreased zebrafish larval whole-body adiposity, and specifically in the head and viscera regions [21]. Additionally, in the same study, HT counteracted the obesogenic effects induced by rosiglitazone. In this study, significant differences were not found in the MFI or adipocyte morphology in visceral fat according to histological evaluation, although slightly lower numbers of adipocytes per µm^2^ were noted in the HF+HT_ST fed group, suggesting the presence of bigger adipocytes that may help in clearing the high lipid load of the diet along with the skeletal muscle. 

In this sense, a significant reduction in plasma FFA levels was noticed in that same HF+HT_ST group compared to the HF_ST one (1.5-fold), supporting altogether the potential fat-lowering effects of HT, as evidenced in mammalian studies [12,14,36]. The hypolipidemic effect achieved by adding HT to the HF diet in fish fed a standard ration was comparable to the effect observed in the reduced ration group, although the combination of HT and restricted feeding did not cause an additional or synergic effect. In any case, the reduction in FFA plasma levels indicated that HT may exhibit protective properties, as elevated circulating FFA levels are typically associated with a negative health impact and are a risk factor for several diseases in humans [41]. Moreover, the gilthead sea bream subjected to a 40% feed restriction showed lower levels of HDL, LDL/VLDL, and TG in plasma than those that were given the standard ration, irrespective of the diet. Lipoproteins synthesis in fish exhibits many similarities to that found in mammals, and their profile in plasma is influenced by dietary lipid load and the type of fatty acid contained, as well as the nutritional status of the fish [1,42]. In this study, these metabolites were not affected by the HT-rich extract, limiting its hypolipidemic effect to FFA levels. Nevertheless, in a previous study by Baba et al. [43], dietary inclusion of an olive leaf extract (rich in polyphenols, such as HT) decreased serum TG levels in rainbow trout, but only in the two groups where the extract concentration was high (i.e., 0.5% and 1% of supplementation), suggesting that, perhaps, the dose of HT used in the current study was insufficient to modify circulating levels of complex lipid forms.

Concerning transcriptional analysis, in the liver, the expression profile of lipid metabolism markers among the four experimental groups was similar across most of the genes evaluated, suggesting higher fatty acid uptake and general turnover when high lipid intake occurs, that is attenuated by the presence of HT. In this regard, fish fed the HFD at a standard ration showed the highest mRNA values in the transcription factors *ppara* and *b*, as well as in the markers associated with fatty acid uptake and transport (*lpl*, *fatp1*, *fabp1*), peroxisomal β-oxidation (*acox1*), and lipolysis (*lipe* and *lipa*). Moreover, regarding the effects on these genes’ levels, and similarly to the observed changes in plasma FFA, the addition of HT to the HF_ST diet was equivalent to lowering the feed ratio, with HT not showing additional effects when administered under restricted conditions. This overall suggests that the impact of HT is evident only when there is a high dietary energy charge.

In terms of fatty acid uptake and transport, the increased mRNA levels of *lpl*, *fatp1* and *fabp1* in the HF_ST group were consistent with the findings documented for blunt snout bream [44,45]. In those studies, both hepatic LPL activity and *lpl* mRNA levels, as well as *fatp* and *fabp* expression, were elevated in the fish fed HFD compared to control fish. Our results suggested that the uptake and transport of fatty acids from TG-rich lipoproteins (chylomicrons and VLDL) by the liver in the HF_ST group increased relative to the other three groups. This observation may help to explain the highest value (although not significant) of lipid content observed in this tissue in terms of proximate analysis. In this sense, the inclusion of the HT-rich extract in the HFD, similarly to diet restriction, downregulated the gene expression of these molecules (namely *lpl*, *fatp1* and *fabp1*), indicating a protective role in preventing the development of diet-induced steatosis, as demonstrated in liver FABP-knockout mice fed a high-saturated fat and high-cholesterol diet [46]. Similar findings in preventing the development of nonalcoholic fatty liver disease were observed in studies involving liver-specific FATPs knockout mice [47,48]. Furthermore, at the gene level, the fatty acid translocase *cd36* was upregulated in the fish fed the HT-supplemented diet (HF+HT), whereas its protein levels were significantly lower compared to those in the fish fed the diet without the extract. This may reflect an increased necessity to produce more transporter protein by upregulating its gene expression, after stimulation by the dietary HT content in this group, although the two processes, gene expression and protein synthesis, are not always regulated in parallel or simultaneously. CD36 is functionally conserved between mammals and teleosts [49,50], and, in the former, hepatic CD36 is known to function as a lipid sensor involved in high-affinity tissue uptake of long-chain fatty acids and under conditions of excessive fat supply [50]. Yet, its role seems to be in terms of regulatory capacity instead of as a quantitative transport mechanism [51]. Interestingly, in CD36-overexpressing transgenic mice, an attenuation of HFD-induced hepatic steatosis was observed [52]. Overall, this suggests that at a transcriptional level, the upregulation of *cd36* together with the downregulation of *lpl*, *fatp1* and *fabp1*, as observed in the HF+HT_ST group, agrees with a protective role of HT in the liver only under a lipid overload context, and for the same reason, this effect was not observed when the additive was given to the restricted group. In contrast to our results, the attenuation of the HFD-induced fat accumulation in the liver by HT, found in the blunt snout bream, was mediated through enhancing fatty acid oxidation, since upregulation of *ppara*, *acox* and *cpt1* was observed in that species [19]. Nevertheless, the expression of these genes was lower in the gilthead sea bream fed the extract compared to the HF-fed fish at a standard ration in the present study. In fact, fatty acids are natural and important ligands for the activation of PPARs [53,54]. Finally, also in those same fish, the parallel increase in lipolysis markers (*lipe* and *lipa*), along with the highest mRNA levels of *fasn*, denoted enhanced lipid turnover in this tissue. 

On the other hand, in white muscle, a pattern similar to that of lipid content (i.e., highest in the HF+HT_ST group) was observed at a transcriptional level with *acox1*, which is the first step and the key enzyme of the peroxisomal fatty acid β-oxidation process [55]. Although it is known that lipid catabolism-related enzymes and pathways are similar in fish and mammals, the division of mitochondria and peroxisomes in fish differs from that of mammals [1]. In fact, peroxisomes only partially contribute to β-oxidation, since they are mainly involved in the shortening of very long-chain fatty acids (C_20_ or more), which are then transported into the mitochondria for complete oxidation and ATP production [55]. In this aspect, the results of this study, in terms of gene expression of the mitochondrial β-oxidation markers (*cpt1a* and *hadh*), did not reveal a parallel increase with that of *acox1* in the muscle of HF+HT_ST fish, suggesting incomplete oxidation of fatty acids and their subsequent accumulation, in agreement with the higher percentage of fat content obtained by the proximate analysis in that group. In terms of fatty acid uptake, the gene expression of *cd36* was significantly reduced in fish fed with the extract and even more under the restricted feeding regime, which could be attributed to a compensatory feedback mechanism in response to lipid accumulation. Indeed, the regulation of fatty acid transporters seems to be very complex and tissue-specific. For instance, the expression of *cd36* in the muscle of salmonids has been reported to either rise or stay unchanged with fasting [49,56]. Conversely, *fatp1* expression was not affected in muscle in the present study, indicating distinct roles between these two carriers in terms of fatty acid uptake. This differential response has been also previously reported in Atlantic salmon (*Salmo salar*) fed with a vegetable oil diet [57] and in myotubes of the same species exposed to insulin [49]. In any case, transcripts for five and six different *fatp* genes have already been found in mice and humans, respectively, with different tissue expression patterns [57], and all of the proteins responsible for this type of fatty acid transport have only just begun to be revealed in fish. Furthermore, in addition to entering into the cells by a membrane-bound protein such as the ones mentioned, uptake of fatty acids could also be taking place by passive diffusion [58], thus leaving some caveats on the total impact of feeding an HT-supplemented diet in fish.

Indeed, in adipose tissue, in support of elevated fatty acid oxidation, the expression of the β-oxidation marker *hadh* was highest in the animals from the HF+HT group when fed the standard ration. Although the peroxisome oxidation marker *acox1* was downregulated in the fish fed the HT extract, the change was not statistically significant. Overall, interpreting these results is complicated, but it appears that at a transcriptional level, mitochondrial peroxidation could be activated by HT but not peroxisomal fatty acid β-oxidation in the adipose tissue. In fact, since the expression of the rest of the genes analyzed was not significantly modified, it can be suggested that lipid metabolism in this tissue is less affected by dietary HT. Furthermore, as gene expression, protein expression, and activity levels are not always equally regulated, it is difficult to have the whole picture of the fatty acid oxidation processes ongoing in the presence of HT to draw more solid conclusions. 

In addition, it has been evidenced that fat accumulation in tissues can lead to oxidative stress, due to an enhanced rate of lipid oxidation [32,59]. Muscle homogenates from rainbow trout and European sea bass fed a HFD showed more susceptibility to lipid peroxidation than those from fish fed low-fat diets [60]. This is attributed to the fact that mitochondria and peroxisomes are recognized as two primary sources of endogenous ROS production that can later inflict direct damage to lipids (peroxidation), among other cell components [61]. Specifically, excessive peroxisomal ROS production can trigger cell death mediated by mitochondria [62], but a protective effect of HT against mitochondrial dysfunction induced by oxidative stress has been demonstrated in C2C12 myocytes [63]. In our work, contrarily, HT supplementation led to increased MDA and 4-HNE levels in white muscle (1.22-fold when comparing the standard fed groups), but not in liver. These secondary end-products are particularly produced after the decomposition of arachidonic acid and larger polyunsaturated fatty acids [61]. In this context, the disrupted structure of mitochondria, along with impaired activity of membrane-anchored enzymes, such as CPT1, has been also proposed as one of the consequences of the lipid peroxidation process [64]. Similarly, previous studies by Lu and co-workers [7,65] demonstrated the loss of cristae and impaired chain enzyme activities of mitochondria in blunt snout bream fed with a HFD. This could provide a possible explanation for the observed increased lipid accumulation and peroxidation in the white muscle of the HF+HT_ST fed gilthead sea bream, as well as the absence of a parallel upregulation of peroxisome and mitochondrial markers in this tissue and group.

Finally, despite the limited availability of established fish cell lines, researchers have widely used in vitro models of primary cell cultures from different fish species for lipid metabolism studies in a more controlled environment [29,30,66]. In our study, the high dose of HT (100 µM) promoted an increase in the viability of adipocytes, irrespective of the diet. In agreement with our results, the same dose of HT also induced increased cell viability after 24 h of exposure in primary cultured day-5 adipocytes from rainbow trout [21]. Conversely, this compound at several doses (0–200 µM) did not exhibit any effect [17] or even decreased [67] viability in the mouse 3T3-L1 adipocyte cell line; however, it has been also linked to pro-apoptotic activity in several mammalian cancer cell lines (reviewed by [11]) and primary human visceral preadipocytes [18] through different signaling pathways. Regarding lipid accumulation, although the values obtained were generally higher in adipocytes from the fish fed with the HF+HT diet than in those from the HF diet-fed fish, in agreement with the in vivo histological data, the overall pattern observed across the different experimental treatments was consistent between both dietary groups. In this sense, exogenous HT treatment exhibited a dose-dependent effect mitigating the obesogenic condition induced by the lipid mixture, and the highest dose of HT (100 µM), when co-incubated with lipid mixture, significantly lowered lipid content compared to the adipocytes with only lipids, returning the levels to those compared to control adipocytes, regardless of the feeding diet of the fish. These findings support the potential of this polyphenolic antioxidant to protect against fat accumulation, modulating the adipocyte differentiation stage. Identical results were obtained in primary cultured adipocytes of rainbow trout when incubated with the same dose of HT and at the same time with either lipid mixture or the thiazolidinedione rosiglitazone known for its pro-adipogenic effects [21]. In mammalian models, a similar inhibitory effect in adipocyte differentiation and lipid content was also reported in C3H10T1/2 preadipocytes exposed to a lower dose (25 µM) of HT but for a longer period (7 days) [68]. Moreover, not only the duration of the treatment but also the timing of culture development appears to be important. Drira et al. [17] found that the decrease in lipid content caused by HT at 100 and 150 µM was only observed in the early stage of differentiation (0–2 days) of 3T3-L1 cells, rather than in the middle (2–4) and late (4–6) stages. According to this, it cannot be ruled out that a prolonged exposure of gilthead sea bream adipocytes to HT could have resulted in a more pronounced anti-obesogenic effect upon the lipid mixture-stimulated fat accumulation.

## 5. Conclusions

To sum up, after 8 weeks of feeding trial, the inclusion of an extract rich in HT (0.52 g HT/kg feed) into a HFD led to a decrease in plasma FFA levels, a downregulation of the hepatic mRNA levels of markers related to fatty acid uptake and transport, and a decrease in the protein levels of the fatty acid transporter CD36 in the liver, suggesting a protective role of HT in preventing the development of diet-induced steatosis, as supported by the observed tendency to reduce hepatic fat accumulation. Conversely, in white muscle, the animals fed with the diet incorporating the additive showed an increase in lipid content and peroxidation, suggesting fat recruitment to this tissue and an enhanced rate of lipid oxidation. In addition, the present work revealed that, in vitro, HT at a high dose increases adipocyte viability and exhibits a dose-dependent effect towards mitigating the accumulation of intracellular fats induced by the lipid mixture condition. On the whole, although additional research should be conducted, particularly in assessing how the observed increase in muscle lipid content affects the nutritional quality and sensory attributes of the fish fillet, the present data support the positive use of HT as a feed additive in gilthead sea bream to mitigate the adverse effects associated with a HFD. The use of a HFD including HT would be useful for feeding the fish during the periods of highest energy demand and growth, such as summer. In fact, besides the healthy metabolic properties shown in the present study for this diet, we have very recently demonstrated [22] that supplementing a HFD with HT can improve the growth capacity of the musculoskeletal system of gilthead sea bream, making this polyphenol a promising additive in functional diets for this species. 

## Figures and Tables

**Table 1:** Composition of the experimental diets. A high-fat (HF) diet alone or supplemented with hydroxytyrosol (HF+HT). The HT was included in the formulation at a dose of 0.52 g HT per kg of feed, and was provided by a commercial extract HIDROX^®^ (Oliphenol LLC., Hayward, CA, USA). This extract is obtained from olive juice and contains > 12% of simple and total polyphenols, from which HT represents 3.136%.

	HF	HF+HT
**Ingredients (%)**		
Corn gluten	3.80	3.80
Wheat gluten	20.00	20.00
Fava beans	8.00	8.00
Soya concentrate	25.00	25.00
Fish oil	9.98	9.98
Fish meal	15.00	15.00
Rapeseed oil	10.14	10.14
Yttrium premix	0.10	0.10
Phosphate	1.04	1.04
Vitamin mineral premix	0.44	0.44
Wheat	6.50	4.85
HIDROX^®^	0.00	1.66
**Composition (%)**		
Dry matter	93.0	93.0
Moisture	7.0	7.0
Crude protein	46.8	46.7
Crude fat	24.0	24.2
Ash	5.4	5.6
Crude fiber	1.9	1.8
Starch	8.8	7.8

**Table 2:** Mesenteric fat index (MFI) and lipid content in the liver and white muscle (WM) of gilthead sea bream fed with the experimental diets. A high-fat diet alone (HF) or supplemented with (HF+HT) hydroxytyrosol (0.52 g HT per kg feed) at standard (ST) (3% total fish weight/tank) or restricted ration (RE) (40% reduction) for 8 weeks.

	HF_ST	HF_RE	HF+HT_ST	HF+HT_RE	D	R	D*R
**MFI**	0.86 ± 0.10	0.68 ± 0.07	1.01 ± 0.16	0.72 ± 0.13	0.435	0.086	0.654
**Liver lipid (%)**	17.08 ± 1.54	16.64 ± 0.66	13.57 ± 1.51	16.73 ± 1.23	0.202	0.307	0.178
**WM lipid (%)**	6.48 ± 0.52 **^a^**	7.89 ± 0.91 **^ab^**	10.26 ± 1.14 **^b^**	7.01 ± 0.90 **^ab^**	0.115	0.311	**0.013**

Data are shown as mean ± SEM (n = 3 tanks; n = 10 fish for lipid content). Statistical differences are indicated in three components: diet (D), ration (R) and interaction (D*R), using two-way ANOVA (*p* < 0.05, shown in bold). Comparisons among groups were analyzed by a Tukey’s post hoc test when the interaction between the two factors was significant, and significant differences are indicated by different letters (*p* < 0.05). MFI: mesenteric fat index (mesenteric fat weight/body weight) × 100.

**Table 3:** Plasma parameters of gilthead sea bream juveniles fed with the experimental diets. A high-fat diet alone (HF) or supplemented with (HF+HT) hydroxytyrosol (0.52 g HT per kg feed) at standard (ST) (3% total fish weight/tank) or restricted ration (RE) (40% reduction) for 8 weeks.

	HF_ST	HF_RE	HF+HT_ST	HF+HT_RE	D	R	D*R
**Glucose (mg/dL)**	96.78 ± 3.09	91.99 ± 3.08	97.41 ± 3.04	97.54 ± 1.68	0.269	0.404	0.378
**FFA (mg/dL)**	6.42 ± 0.47 **^a^**	4.45 ± 0.42 **^b^**	4.28 ± 0.23 **^b^**	4.98 ± 0.38 **^b^**	**0.047**	0.109	**0.002**
**HDL (mg/dL)**	289.81 ± 13.48	224.10 ± 16.33	264.41 ± 7.27	237.63 ± 11.74	0.633	**<0.001**	0.124
**LDL/VLDL (mg/dL)**	81.33 ± 13.98	48.93 ± 5.44	87.46 ± 7.78	59.08 ± 5.10	0.390	**0.003**	0.831
**TG (mg/dL)**	335.06 ± 21.06	276.00 ± 14.09	325.17 ± 28.63	293.17 ± 18.12	0.871	**0.049**	0.546

Data are shown as mean ± SEM (n = 10 fish). Statistical differences are indicated in three components: diet (D), ration (R) and interaction (D*R), using two-way ANOVA (*p* < 0.05, shown in bold). Comparisons among groups were analyzed by a Tukey’s post hoc test when the interaction between the two factors was significant, and significant differences are indicated by different letters (*p* < 0.05). FFA: free fatty acids; HDL: high-density lipoproteins; LDL: low-density lipoproteins; VLDL: very low-density lipoproteins; TG: triglycerides.

**Table 4:** Lipid peroxidation (LPO) levels (nmol MDA + 4-HNE/mg protein) in liver and white muscle (WM) of gilthead sea bream juveniles fed with the experimental diets. A high-fat diet alone (HF) or supplemented with hydroxytyrosol (HF+HT) (0.52 g HT per kg feed), at a standard (ST) (3% total fish weight/tank) or restricted ration (RE) (40% reduction) for 8 weeks.

	HF_ST	HF_RE	HF+HT_ST	HF+HT_RE	D	R	D*R
**Liver LPO**	0.994 ± 0.09	1.039 ± 0.17	0.927 ± 0.12	0.814 ± 0.10	0.230	0.779	0.513
**WM LPO**	0.050 ± 0.005	0.039 ± 0.005	0.061 ± 0.008	0.068 ± 0.007	**0.004**	0.786	0.164

Data are shown as mean ± SEM (n = 10 fish). Statistical differences are indicated in three components: diet (D), ration (R) and interaction (D*R), using two-way ANOVA (*p* < 0.05, shown in bold).

## Data Availability

The data presented in this study are available in the current article and its corresponding Supplementary Materials.

## Supplementary Materials

The following supporting information can be downloaded at: https://www.mdpi.com/article/10.3390/antiox13040403/s1, Table S1: Primers used in the real-time quantitative PCR analyses.
