# Supplementary material for: Impact of Hydroxytyrosol-Rich Extract Supplementation in a High-Fat Diet on Gilthead Sea Bream (Sparus aurata) Lipid Metabolism
Source: Antioxidants (Basel). 2024 Mar 27;13(4):403. doi: 10.3390/antiox13040403 (PMC11047642; doi:10.3390/antiox13040403)
Supplement: Supplementary file 1 [file antioxidants-13-00403-s001.zip › antioxidants-2912286-supplementary.pdf]

Table S1. Primers used in the Real-Time quantitative PCR analyses.

Type	Gene	Primer sequences (5'–3')	Ta (ºC)	Accession number
Reference	*rps18*	**F**: GGGTGTTGGCAGACGTTAC<br>**R**: CTTCTGCCTGTTGAGGAACCA	60	AM490061
	ef1a	**F**: CTTCAACGCTCAGGTCATCAT<br>**R**: GCACAGCGAAACGACCAAGGGGA	60	AF184170
	rpl27a	**F**: AAGAGGAACACAACTCACTGCCCCAC<br>**R**: GCTTGCCTTTGCCCAGAACTTTGTAG	68	AY188520
Lipid metabolism	*ppara*	**F**: TCTCTTCAGCCCACCATCCC<br>**R**: ATCCCAGCGTGTCGTCTCC	62	AY590299
	pparb	**F**: AGGCGAGGGAGAGTGAGGATGAGGAG<br>**R**: CTGTTCTGAAAGCGAGGGTGACGATGTTTG	69	AY590301
	pparg	**F**: CGCCGTGGACCTGTCAGAGC<br>**R**: GGAATGGATGGAGGAGGAGGAGATGG	66	AY590304
	lpl	**F**: GAGCACGCAGACAACCAGAA<br>**R**: GGGGTAGATGTCGATGTCGC	60	AY495672
	cd36	**F**: CCTTGAGGTTTGCCAAGAGGA<br>**R**: ATCCAACGCAGCCGTCTCA	60	XM_030440140
	fatp1	**F**: CAACAGAGGTGGAGGGCATT<br>**R**: GGGGAGATACGCAGGAACAC	60	XM_030407649
	fabp1	**F**: TTCTCACTTTACCATTGCGGC<br>**R**: AGTTCATCAGGGAGACCAATCG	60	XM_030418150
	acox1	**F**: GGAGATTTCCTACAGGCTGGAC<br>**R**: CTGTGGCGTTTAGGGGTGAG	58	XM_030420563
	cpt1a	**F**: GTGCCTTCGTTCGTTCCATGATC<br>**R**: TGATGCTTTATCTGCTGCCTGTTTG	60	XM_030426201
	hadh	**F**: GAACCTCAGCAACAAGCCAAGAG<br>**R**: CTAAGAGGCGGTTGACAATGAATCC	60	XM_030431227
	fasn	**F**: TGGCAGCATACACACAGACC<br>**R**: CACACAGGGCTTCAGTTTCA	60	XM_030399576
	atgl	**F**: GTGCTTCAGTCCTGGATGTCTTC<br>**R**: AGCCTTGCAGGTCCATGTTGA	60	XM_030424590
	lipe	**F**: GCTTTGCTTCAGTTTACCACCATTTC<br>**R**: GATGTAGCGACCCTTCTGGATGATGTG	60	XM_030393851
	lipa	**F**: TACTACATCGGACACTCTCAAGGAAC<br>**R**: GTGGAGAACGCTATGAATGCTATCG	60	JQ308831

F: forward; R: reverse; Ta: annealing temperature; *rps18:* 40S ribosomal protein S18; *ef1a*: elongation factor 1-alpha; *rpl27a*; ribosomal protein L27a; *ppara*: peroxisome proliferator activated receptor alpha; *pparb*: peroxisome proliferator activated receptor beta; *pparg*: peroxisome proliferator activated receptor gamma; *lpl*: lipoprotein lipase; *cd36*: cluster of differentiation 36; *fatp1*: long-chain fatty acid transport protein 1; *fabp1*: fatty acid binding protein 1; acox1: acyl-CoA oxidase 1; *cpt1a*: carnitine palmitoyltransferase 1a; *hadh*: hydroxyacil-CoA dehydrogenase; *fasn*: fatty acid synthase; *atgl*: adipose triglyceride lipase; *lipe:* lipase E, hormone-sensitive lipase; *lipa*: lipase A.
